# Stripping Mechanism of Surfactant System Based on Residual Oil on the Surface of Sand-Conglomerate Rocks with Different Grain Size Mineral Compositions

**DOI:** 10.3390/molecules29061278

**Published:** 2024-03-13

**Authors:** Yuanyuan Wang, Daigang Wang, Chao Ding, Jing Li, Shengdong Jiang

**Affiliations:** 1 Key Laboratory of Enhanced Oil & Gas Recovery of Ministry of Education, Northeast Petroleum University, Daqing 163318, China; 2 National Key Laboratory of Petroleum Resources and Engineering, China University of Petroleum, Beijing 102249, China; 3 Development Division, Xinjiang Oilfield Co., Ltd., Kelamayi 457001, China; 4 Yumen Oilfield Co., Ltd., Yumen 735200, China; 5 College of Chemistry Chemical Engineering, Daqing Normal University, Daqing 163111, China

**Keywords:** sand-conglomerates, surfactant, mineral adsorption, oil-stripping mechanism

## Abstract

During the development of a sand-conglomerate reservoir, there is a huge variation in rock grain size and different åmineral compositions of different-sized sand grains. The mineral composition and microstructure of the rock both have an impact on the characteristics of the remaining oil in the reservoir. The stripping mechanism of a surfactant system on sand-conglomerate surface crude oil with varied grain size minerals was explored in this paper. Sand-conglomerate was classified and analyzed to determine their wettability and stripping oil effects. The optimization of the surfactant solution system and molecular dynamics simulation revealed the surfactant stripping mechanism on crude oil on distinct sandstone minerals. The results of the study showed that montmorillonite minerals are more readily adsorbed by surfactants. The crude oil within them is more likely to compete for adsorption and to be stripped off, and then extracted with the recovery fluid. The surfactant solution system can increase the hydrophilicity of the rock surface, make the crude oil on the rock surface shrink and gather, and enhance the transportation ability of the displacement fluid. And the emulsification seals part of the pore in the reservoir, increases the displacement pressure, and improves the overall wave volume. The results of this paper are of great significance for the efficient development of sand-conglomerate reservoirs.

## 1. Introduction

Crude oil is a non-renewable energy source, but it is also the raw material for a large number of chemical products. Despite the development of a variety of alternative energy sources in recent years, the worldwide need for crude oil is still continuing to grow [1]. As a result, working to improve the theoretical and technological levels of oil extraction is extremely important [2,3].

Sand-conglomerate reservoirs are challenging to exploit and have a complicated residual oil distribution because of their distinct rock-forming properties, which also cause significant reservoir non-homogeneity and complex mineral composition [4]. Sand-conglomerate cementation is structurally complex and highly heterogeneous, and traditional fractal dimensions are insufficient to fully characterize the pore space [5,6]. Zhou, Y. et al. [7] established a model based on the Pia Intermingled Fractal Units (IFU), taking into account the variation factors of tortuality and boundary layer thickness. They also described the sand-conglomerate reservoir of the Karamay Formation in the northwest Junggar Basin. The sand-conglomerate oil reservoir’s mineral composition and grit particle size, however, cannot be guaranteed by the fractal theory alone. Gong, Q. et al. [8] used geological and cable logging data, as well as dynamic production data, to investigate the non-homogeneity of sand-conglomerate reservoirs in the Qaidam Basin and its impact on residual oil distribution. At the same time, the physical analysis of sand and sand-conglomerate using scanning electron microscopy, casting thin sections, and three-dimensional seismic data is an effective method for accurately determining the type of reservoir lithology as well as the physical properties of rock [9]. In summary, the development of sand-conglomerate reservoirs involves classifying the size of sand grains and the composition of minerals to identify methods for enhancing the recovery. Conventional water-driven development in sand-conglomerate reservoirs is likely to result in the formation of dominant seepage channels and reduced oil production due to the heterogeneity of reservoirs. Hence, the enhancement of oil production efficiency necessitates the integration of surfactants, polymers, and other oil displacement mechanisms in sand-conglomerate reservoirs [10,11,12,13]. Polymers have the capacity to enhance the viscosity of the replacement fluid, leading to a significant decrease in the ratio of oil flow. This, in turn, enhances the effectiveness of water-driven ripples that are irregularly spread out as a result of the non-uniform characteristics of reservoirs. The application of a versatile composite oil displacement system, along with surfactant to enhance its microscopic oil-stripping efficiency, has been demonstrated to be effective in the development of sand-conglomerate reservoirs. Lu, J. et al. [14] established a technique for forecasting the critical velocity of surfactants by examining the connection between microemulsion viscosity and critical velocity. They argued that optimizing the microemulsion viscosity can enhance the critical velocity, thereby ensuring a steady flow of the microemulsion through the porous medium.

Sand-conglomerate reservoirs exhibit significant variation in the size of rock particles. There exists a specific relationship between the quantity of remaining oil and the size of sand particles. Consequently, the amount of remaining oil can be effectively aligned with the surfactant system that is most suitable for the various sand-conglomerate types [15,16]. The driving system involves competitive adsorption, where crude oil is adsorbed onto the surface of the rock. This process is closely linked to the stripping effect of crude oil. An investigation into the recovery enhancement process involves evaluating the adsorption of crude oil and the driving fluid on the rock surface [17]. Researchers of significance have examined the relationship between the driving force and crude oil by creating mathematical and physical models of surfactants and polymers that drive oil. They have also simulated the behaviors of various combinations of polymers and surfactants and uncovered certain mechanisms of improved oil recovery in composite driving systems [18,19,20]. Nevertheless, the attainment of these outcomes is mostly dependent on indoor trials, with a greater emphasis on qualitative rather than quantitative research. However, further exploration in theoretical research is still necessary. This research focuses on the significant heterogeneity of the stratigraphy of sand-conglomerate. Natural cores were chosen and subjected to grain size classification and division. Furthermore, the mineral compositions of various grain sizes were studied. By combining surfactant stripping oil experiments with core replacement experiments, the recovery effect of surfactants with different types of sand-conglomerate reservoirs was investigated. The study examined the adsorption and retention properties of surfactants on various minerals of different particle sizes. By conducting experiments and utilizing molecular dynamics simulation methods, this research revealed the effects of surfactants and crude oil on different types of minerals. The results in this paper can offer theoretical assistance in the efficient exploitation of sand-conglomerate oil reservoirs.

## 2. Results and Discussion

### 2.1. Wettability and Oil-Stripping Effect of Surfactant Solutions on Minerals with Different Particle Sizes

**Figure 1:**
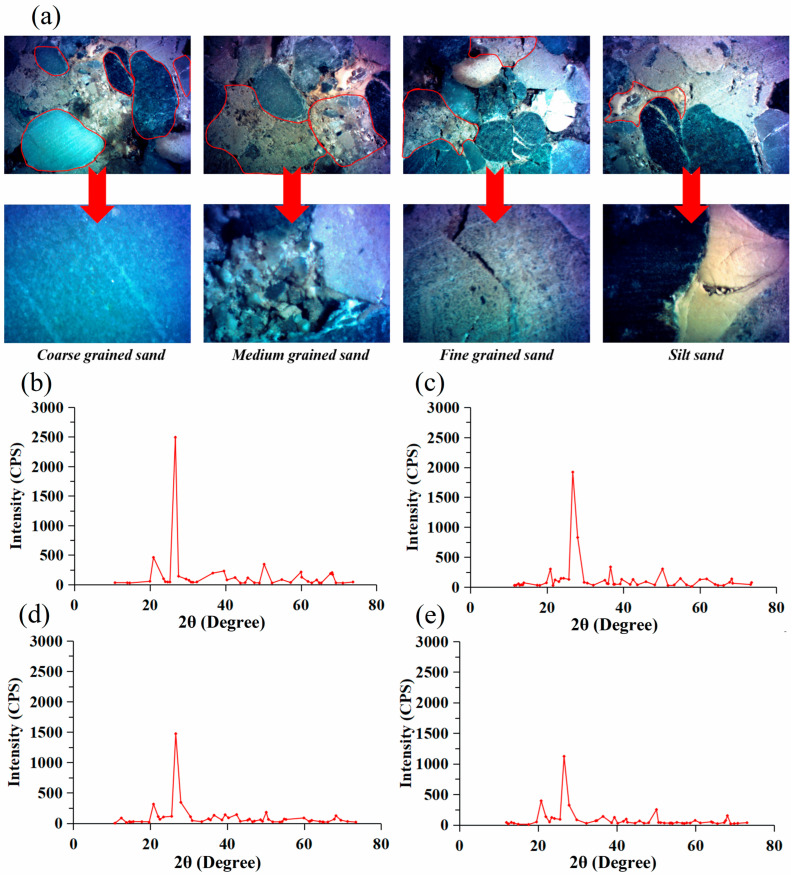
Mineral characterization of conglomerates with different particle sizes. (**a**) The microscopic features of various conglomerate types under the microscope; (**b**–**e**) the equivalent X-ray diffraction data for coarse, medium, fine, and silt sand, respectively.

An investigation is required to examine the impact of surfactant solutions on the minerals found in conglomerate formations, as these minerals have varying compositions. The natural conglomerates were sorted and crushed for the investigation. Figure 1 illustrates the mineralogical characteristics of conglomerates with different particle sizes.

The conglomerate is classified into coarse-grained sand, medium-grained sand, fine-grained sand, and silt sand based on the varying sizes of its particles. Coarse-grained sands are mostly composed of quartz, have pores that are relatively compact, and have a low concentration of crude oil. Medium- and fine-grained sands are characterized by their particle size and composition of feldspars and clay minerals. The grain size of silt sand, which is predominantly composed of clay particles, is much smaller. The wettability of fluid was assessed on various sizes of rock fragments by pulverizing different conglomerates, using the research methods outlined previously.

**Figure 2:**
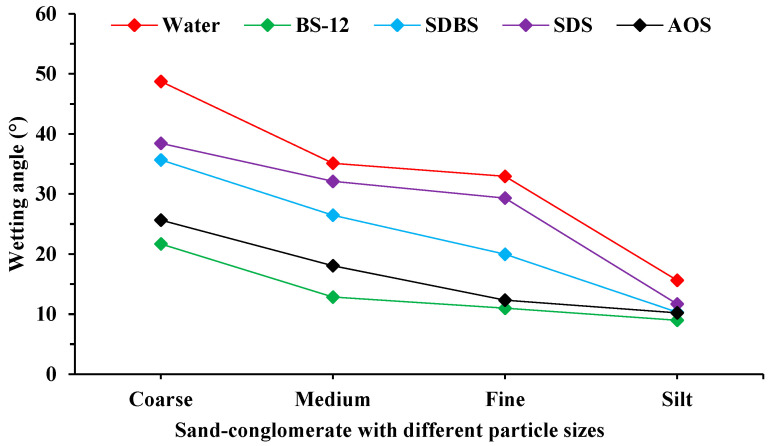
The fluctuation in oil-stripping effectiveness of several surfactants on diverse sand-conglomerate types.

Figure 2 illustrates that the contact angle for surface wetting of coarse-grained sand is greater, with water exhibiting a wetting angle of 48.75°. This suggests that coarse-grained sand has a higher affinity for lipids. The wettability of medium-grained sand and fine-grained sand is similar, with water having wetting angles of 35.12° and 32.94°, respectively. The presence of clay minerals in medium- and fine-grained sands leads to a significant level of hydrophilicity. The wetting angle of water on the silt sand is significantly reduced to 15.64°, suggesting a high level of hydrophilicity and a greater presence of clay minerals. The application of surfactant reduces the wetting angle of various types of sand-conglomerate to varying extents. This indicates that the surfactant system can be successfully absorbed onto the surface of the rocks, altering their ability to interact with fluids in the reservoir. Surfactants are more effective in coarse, medium, and fine-grained sands. Surfactant solutions have limited effectiveness in silt sand with significant clay concentration. It enhances the hydrophilicity of the rock surface, causing the original adsorption of crude oil to contract and aggregate. Ultimately, this leads to the detachment and transportation of the crude oil from the rock surface. Furthermore, it is crucial to determine the efficacy of surfactants in stripping oil from sand-conglomerates of varying grain sizes when developing conglomerate reservoirs, as different surfactant systems exhibit distinct mineral adsorption capabilities.

**Figure 3:**
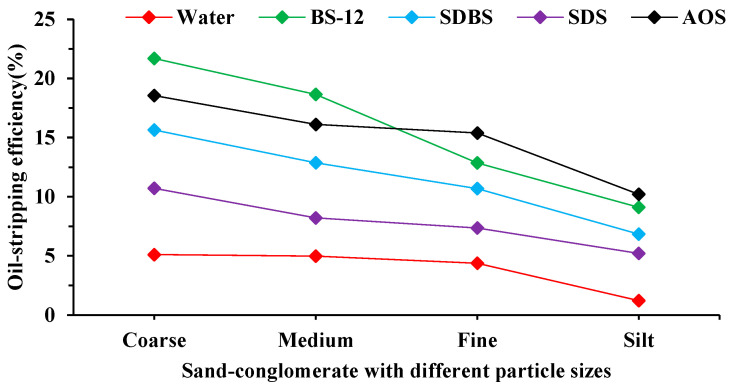
Variation in oil-stripping efficiency of different surfactants on different types of sand-conglomerates.

Figure 3 demonstrates that various surfactant solutions significantly impact the oil-stripping effect of distinct conglomerate fractions. The betaine surfactant system demonstrates the most effective oil-stripping effect for coarse-grained sand powder and medium-grained sand powder, with percentages of 21.69% and 18.65%, respectively. The AOS surfactant system demonstrated superior oil-stripping efficacy for fine-grained sand powder and chalk sand powder. This indicates that various surfactants exhibit distinct oil-stripping effects on different conglomerate components. The oil-stripping efficiency was lowest under water, with a maximum of only 5.11%. The conglomerate involved in this study is mainly dominated by coarse-grained and medium-grained sands, which are the main storage of crude oil. Consequently, the BS-12 solution was chosen as the initial focus of the investigation. The mineral compositions in conglomerate reservoirs exhibit notable distinctions from typical sandstone reservoirs, necessitating the selection of distinct mineral types for modeling conglomerate rocks. Consequently, the core replacement experiment was conducted, yielding the water content and recovery rate change curves for various types of mineral rocks.

**Figure 4:**
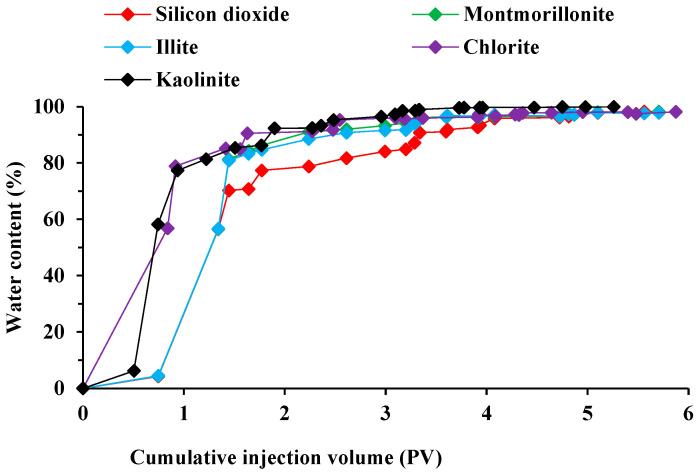
Water content variation curves for several mineral rock types.

**Figure 5:**
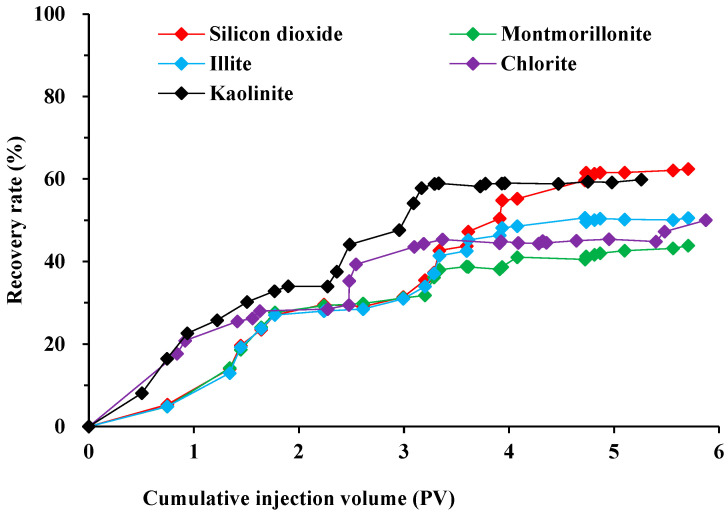
Recovery rate variation curves for several mineral rock types.

The comparison of Figure 4 and Figure 5 reveals distinct variations in the ability of various surfactants to repel oil on rocks with different mineral compositions. Unlike the oil-stripping experiment depicted in Figure 3, rocks predominantly composed of silica exhibit superior oil-repelling properties owing to the impact of their pore structure. The recovery after the surfactant system was up to 62.41%. For cores containing kaolinite and illite, the recoveries after surfactant replacement were 59.86% and 50.55%. The analysis revealed that kaolinite and illite exhibited distinct adsorption capacity and wetting reversal capacity towards surfactants, resulting in variations in the final recovery rate. The limited improvement in recovery observed when montmorillonite and chlorite were replaced with a betaine surfactant solution can be attributed to the susceptibility of these minerals to the properties of the injected liquids. The swelling of the clays in the rocks obstructed certain pore throats, leading to a decrease in the recovery rate.

### 2.2. Adsorption Behavior of Surfactants on Various Minerals

**Figure 6:**
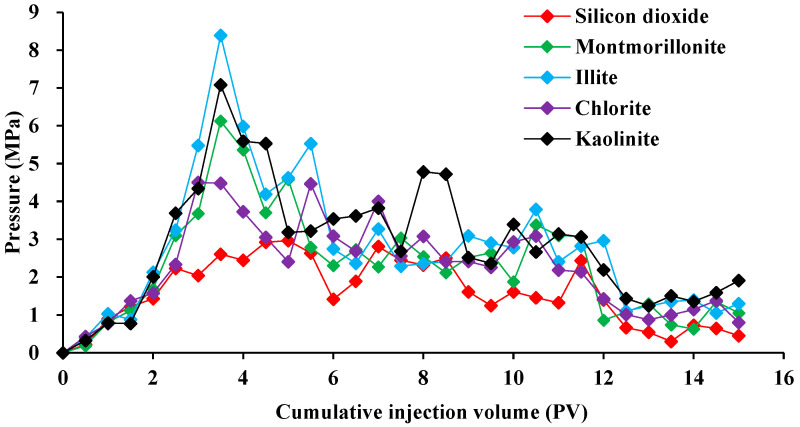
Effect of surfactants on the displacement pressure for several mineral rock types.

**Figure 7:**
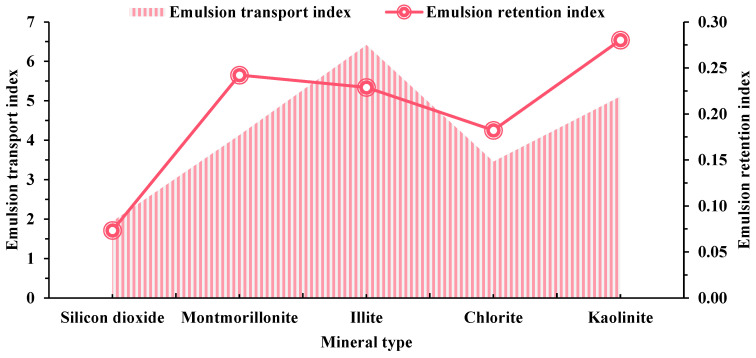
Influence of emulsion transport and retention index for different types of minerals (BS-12).

The reservoir exhibits an intricate pore structure, leading to the emulsification of oil and water phases due to the influence of shear and surfactants present in the porous medium. Consequently, the production of oil is hindered by the combined effects of emulsification and adsorption. Hence, to investigate the adsorption retention behavior of surfactants on various minerals, the experimental approach described in Section 2.3 was employed to ascertain the variation pattern of displacement pressure under different circumstances. The experimental results are shown in Figure 6 and Figure 7.

The displacement pressure of the BS-12 surfactant system exhibited substantial variations in cores containing different mineral kinds. The initial injection pressure was insufficient, but after the injection was mixed, the displacement pressure increased to a peak of 8.39 MPa, suggesting that the emulsion was formed within the porous medium. When comparing different types of rocks, silica has the lowest replacement pressure, measuring only 2.97 MPa. This is because the liquid solution containing surfactant is absorbed into the interior of the rock.

The emulsion transport and retention indices reflect alterations in the surfactant solution’s ability to transport and retain substances, resulting from the combined impact of surfactant emulsification and adsorption. Surfactant retention is low in silica minerals and high in rocks that contain kaolinite and montmorillonite. This indicates that cores with higher concentrations of montmorillonite and kaolinite have a greater affinity for the BS-12 surfactant, resulting in increased retention capacity. When the transport index is elevated, the replacement fluid exhibits enhanced transport capacity, while the ability of the surfactant-induced oil–water emulsion formation is diminished. Consequently, the crude oil is transported in a continuous flow manner within the porous medium, and the displacement pressure remains constant. The findings indicate that cores containing illite and kaolinite have a greater transport index, while silica cores have a lower value. The adsorption of surfactant has an impact on the transport and ability to retain the displacement fluid. Both the adsorption along the stream and the emulsification of surfactant due to shear are influenced by the concentration of surfactant. Additional measurements of the variation in displacement pressure reveal varying amounts of the BS-12 surfactant.

**Figure 8:**
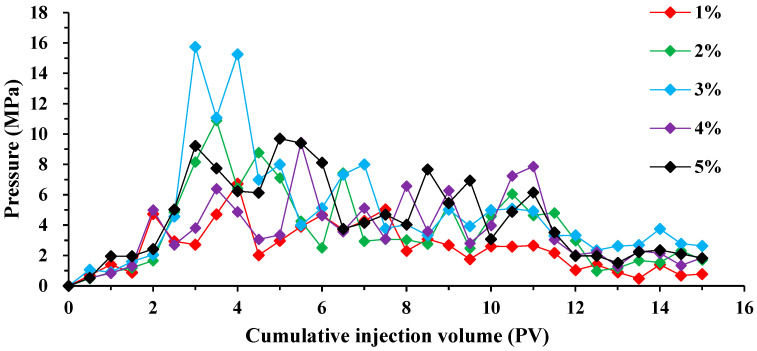
Variation in displacement pressure for different concentrations of BS-12 solution.

Figure 8 illustrates that the injection pressure remains relatively constant during the oil–water co-injection stage. However, the displacement pressure varies as the concentration of BS-12 surfactant increases. Displacement pressures were low at 1% concentration, with a maximum pressure of only 6.42 MPa. Furthermore, when the surfactant concentration grew, there was a substantial rise in pressure, reaching a maximum of 15.75 MPa when the surfactant concentration was 0.3%. The presence of surfactant causes the emulsification of crude oil–water within the rock, resulting in this phenomenon. The variation in shear forces between the oil and water phases as they move through the core leads to the formation of emulsion particles of varied sizes throughout the stripping process. Consequently, the injection pressure does not have a direct relationship with the concentration of surfactant. The impact of varying surfactant concentrations on the transport index and retention index was subsequently estimated.

**Figure 9:**
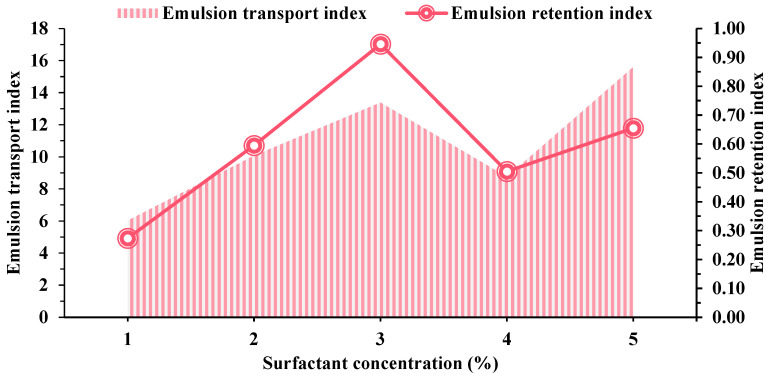
Effect of different surfactant concentrations on transport and retention indexes.

Figure 9 demonstrates that the adsorption and transport abilities of surfactants injected at various concentrations are comparable. Notably, when the surfactant concentration reaches 3%, both the transport and retention capacities are enhanced, suggesting a clear emulsification of oil and water after the surfactant system enters the rock. The emulsified system results in an elevation of the displacement pressure at the depicted stage in Figure 8. Nevertheless, the displacement pressure gradually returns to a stable state during the later stages, implying that the surfactant is progressively absorbed onto the surface of the rock, leading to an increase in transport capacity.

### 2.3. Mechanism of Oil-Stripping by Surfactants on the Surface of Different Conglomerate Minerals

In different types of conglomerate reservoirs, there is a correspondence between the location of residual oil and the mineral type. Surfactants also have different residual oil types and stripping effects within the minerals. Hence, to investigate the microscopic process of removing oil from mineral surfaces using various surfactants, the density distribution functions of crude oil on different mineral surfaces were determined. 

**Figure 10:**
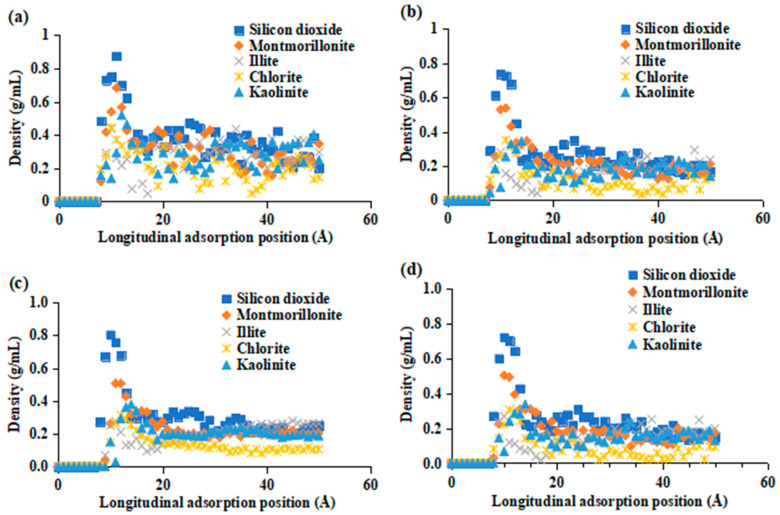
Density distribution of crude oil on mineral surfaces in different surfactants. (**a**) Water and surfactants in (**b**–**d**) are SDBS, SDS, and BS-12, respectively.

Figure 10 illustrates the results of the density distribution of crude oil on the mineral under different surfactants. Various surfactants exhibit varying degrees of adsorption on different minerals, leading to the competitive adsorption of crude oil on the surfaces of these minerals. In general, rocks that contained silica exhibited the highest capacity for adsorption. Additionally, the presence of surfactants resulted in a relatively high density of crude oil. Surfaces containing montmorillonite and kaolinite showed a relatively low capacity for adsorbing crude oil. The reason for this phenomenon is the contrasting charged characteristics on the surfaces of these two mineral kinds. When a surfactant is introduced, montmorillonite readily absorbs a significant quantity of the surfactant on its surface. This absorption process causes the surfactant to dislodge the crude oil, hence decreasing the density of crude oil molecules on the rock surface. Upon comparing several surfactants, it was discovered that BS-12 and SDBS exhibited superior rock modification effects. Furthermore, the presence of surfactants led to a notable decrease in the density of crude oil. The alteration of mineral surfaces with surfactants also impacts the mobility of crude oil by increasing the diffusion coefficient of crude oil molecules inside the oil film. Consequently, this makes the oil film more susceptible to removal. In order to validate this concept, the mean squared displacement (MSD) of crude oil molecules at the specific position on the rock surface was computed in various situations as a function of time.

**Figure 11:**
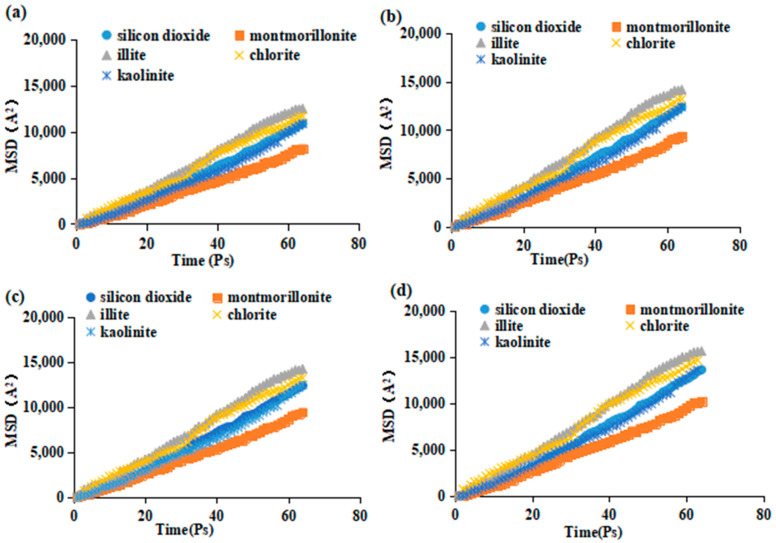
MSD of crude oil molecules on different mineral surfaces as a function of time. (**a**) Water and surfactants in (**b**–**d**) are SDBS, SDS, and BS-12, respectively.

Figure 11 demonstrates that the MSD of crude oil molecules varies notably on various mineral surfaces. Additionally, the inclusion of the surfactant results in a significant increase in the slope of the MSD at the mineral surface location. Furthermore, the emulsifier effectively improves the mobility of the crude oil molecules and enhances the competitive adsorption capacity of oil and water. Comparing the various surfactants, it was found that the movement of crude oil molecules on the mineral surface was greatly improved after the addition of surfactants. This improvement was evident in the increased steepness of the curve representing the MSD versus time. When montmorillonite is present, the capacity for surfactant adsorption is enhanced, leading to more intense competition with crude oil for adsorption. As a result, the crude oil is more prone to being removed and then retrieved with the recovery fluid.

## 3. Experiment and Methodology

### 3.1. Materials and Instruments

The material used in this study includes crude oil, natural cores, artificial cores (300 mm × 300 mm × 4.5 mm), polyacrylamide, deionized water, and surfactants. The crude oil and sand-conglomerate natural core were provided by Xinjiang Oilfield (Kelamayi, China), where the viscosity of the crude oil was 50 mPa·s at 50 °C. The partially hydrolyzed polyacrylamide used in the experiments was produced by Henan Aisen Environmental Protection Co. (Anyang, China). The molecular weight of the polymers used in the experimental process was 25 million, and the solid content was 90.6%. The surfactants include 90% sodium dodecyl benzene sulfonate (SDBS), 60% sodium dodecyl sulfate (SDS), 30% dodecyl dimethyl betaine (BS-12), and 92% sodium alpha-olefin sulfonate (AOS). The sand-conglomerate particles were observed using a PM 6000 electron microscope made by Hengqin Instrument and Equipment Factory in Shanghai, China. The XF3000 Laser Particle Sizer, produced by Xiongfa Instruments in Xiamen, China, was utilized to determine the particle size of powdered rock samples. The wetting angle was measured by model A801S dynamic and static contact angle meter, Kino, Boston, MA, USA. The instrument used in the crude oil displacement experiment is the core experiment displacement system independently developed by Northeast Petroleum University.

### 3.2. Influence of Surfactants on the Wettability and Oil-Stripping Effect of Minerals with Different Particle Sizes

#### 3.2.1. Rock Wettability Experiment

The wettability of rock influences the surface characteristics of the stratigraphic porous media, determining its oleophilic and hydrophilic properties. The complex pore structure of sand-conglomerate rock, the particle size of the conglomerate, and the matrix sandstone portion of the rock also affect the distribution of crude oil within the rock. The mineralogical composition of the rock exhibits clear disparities, which are being examined to assess the impact of various surfactants on the alteration of conglomerate rock’s wettability. It is imperative to categorize the conglomerate rock samples based on their particle size distribution. 

The experimental steps are as follows:

(1) The focal length of the instrument was checked. A specimen with the same thickness as the sample to be tested was selected, and a droplet of surfactant solution with a volume of 4 μL was dropped on the center of its surface, and the camera system was adjusted so that the needle and droplet contours were clearly imaged in the contact angle measuring instrument.
(2) The surface of the rock sample was made into a thin slice, and the surface of the slice was ground flat. The sample was placed on the test bench of the instrument, the height of the sample bench was adjusted, the droplet flow rate was set to 0.5 μL/s, the droplet contacted with the surface of the sample to be tested and disengaged from the needle (the diameter of the needle was 0.9 mm), and a seated drop was formed on the surface of the sample. The formation of the seated drop and the spreading of the seated drop were photographed with a contact angle meter at a speed of not less than 2 frames/s. The test time was 180 s.
(3) Five photographs with contact times ranging from 55 s to 65 s were identified, and the contact angles were calculated by the spherical calculation method, and the average value of the above contact angles was taken as the static contact angle of the samples.

#### 3.2.2. Oil-Stripping Efficiency Experiment

The primary factors influencing the recovery of crude oil are the volume of the replacement fluid and the efficiency of oil stripping [21]. To assess the impact of different surfactants on various sand-conglomerate samples, it is essential to first investigate the oil-stripping effect of these surfactants.

The experimental steps are as follows:

(1) The washed quartz sand was mixed with crude oil in a ratio of 1:6 to make the oil sand, with the mixture aged for 24 h at 60 °C.
(2) The surfactant solution with a mass concentration of 0.5% was mixed with deionized water.
(3) The oil sand (*m*_1_ = 15 g) was placed in the centrifuge tube, and the surfactant was added according to the mass ratio of oil sand/surfactant = 1:2. The samples were put into the centrifuge, which can make the oil sand more compact and achieve the effect of stripping static test.
(4) The mixture in the centrifuge tube of step 3 was filtered, and the filtered oil sand was wrapped and put into a drying oven for 24 h.
(5) The oil sand in step 4 was weighed, and the oil-stripping efficiency was calculated using Equation (1).

(1) $$\eta =\frac{m_{1}-(m_{2}-m_{0})}{m_{1}\times p}$$

where *η* is the oil-stripping efficiency, %; *m*_0_ is the mass of the centrifuge tube, *g*; and *p* is oil content, % ($m_{1}\times p=2.14g$).

#### 3.2.3. Oil Displacement in Different Conglomerate Types by Emulsifier Systems

To elucidate the impact of surfactant solution on sand-conglomerate samples, an experiment investigating the driving effect of an emulsifier system was conducted. The procedural sequence is as follows.

(1) Artificial cores with physical characteristics resembling those of the reservoirs being studied were collected and deposited in a drying oven. Subsequently, the length and dry weight (*m*_0_) of the cores were measured.
(2) The core was soaked with formation water and the weight of the soaked core was measured.
(3) A core full of formation water was put into the core adder. Simulated oil was then pumped into the core at a rate of 0.1 mL/min to push the oil away until the recovered fluid was free of water and the water output was recorded.
(4) Following water-driven dehydration to achieve a water content of 98% at a flow rate of 0.1 mL/min, a surfactant solution with a concentration of 0.3% was injected using a volume of 0.5 PV (pore volume). Subsequent water-driven dehydration was then conducted to observe the variations in oil production, fluid production, and pressure throughout the experiment.
(5) The emulsification regulation system and cores were adjusted.

### 3.3. Adsorption Retention Experiments of Surfactants on Minerals with Different Particle Sizes

**Figure 12:**
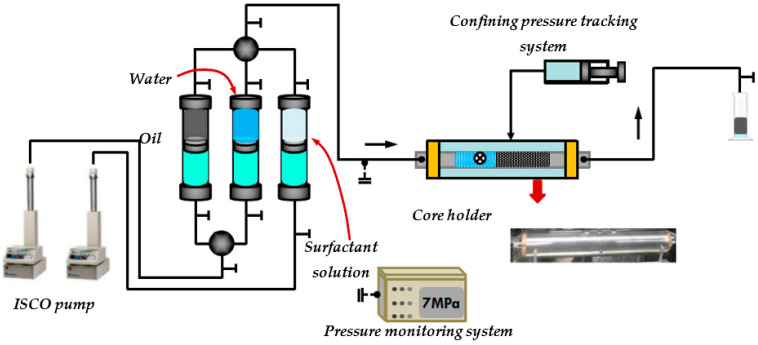
Measuring device for emulsion transportation index and retention index (The arrow indicates the type of solution in the container).

The emulsification capacity in porous media refers to the capability of the emulsion formed by the movement of a surfactant solution in porous media to flow and be transported inside the pores, while also being collected and maintained by the pores. The transport capacity of emulsion in porous media is quantified by the emulsion transport index, whereas the retention capacity of emulsion in porous media is quantified by the emulsion retention index. Two pumps operating at a constant speed of 0.1 mL/min were activated. They were used to inject 0.75 PV of simulated crude oil and 0.75 PV of simulated formation water into the core simultaneously, also at a speed of 0.1 mL/min. The pressure sensor recorded the pressure measurements for each injected volume. The pressure experienced during the injection of 0.5 PV of experimental oil was measured and recorded as *p_wo_*. The outlet valves of the containers containing the surfactant solution and crude oil were simultaneously opened, while the outlet valve of the container containing the formation water was closed. Each substance was injected into the core at a rate of 0.1 mL/min, with an injection volume of 5 PV. The pressure value of the pressure sensor was recorded at every 0.125 PV injection. The pressure during the injection of 4.5 PV to 5 PV of experimental oil was averaged and recorded as *p_em_*. The outlet valves of the containers holding the formation water and crude oil are opened and the outlet valve of the surfactant solution container is closed. The core was filled with 1.5 PV of fluid. The pressure sensor pressure was recorded for every 0.125 PV of the experimental oil injected. The pressures during the injection of 1 PV to 1.5 PV of experimental oil were averaged and recorded as *p_ewo_*. The experimental procedure is illustrated in Figure 12.

The emulsion transport index was calculated according to Equation (2).

(2) $$T_{ei}=\frac{p_{wo}\mu_{s}}{p_{em}\mu_{w}}$$

where *T_ei_* is the emulsion transport index; *p_wo_* is the average pressure of oil and water seepage, MPa; *p_em_* is the average pressure of emulsion seepage, MPa; *μ_s_* is the apparent viscosity of chemical oil repellent system solution at the target reservoir temperature, mPa·s; *μ_w_* is the apparent viscosity of brine at the target reservoir temperature, mPa·s.

The emulsion retention index was calculated according to Equation (3).

(3) $$R_{ei}=\frac{p_{ewo}}{p_{wo}}$$

where *R_ei_* is the emulsion retention index; *p_ewo_* is the average pressure of oil–water two-phase seepage after emulsion generation, MPa.

### 3.4. Molecular Modeling of Surfactants with Different Conglomerate Minerals

**Figure 13:**
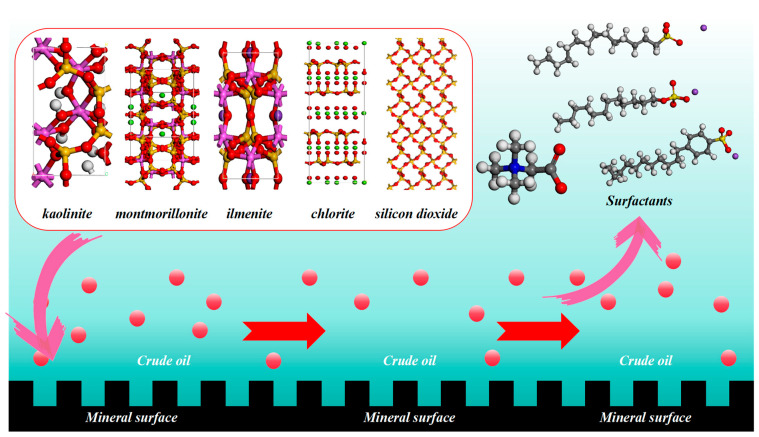
Molecular model of competitive adsorption.

Multiple investigations have demonstrated significant variations in the adsorption characteristics of surfactants on various mineral components and their capacity to alter surface wettability. Consequently, the surfactants exhibit varying capacities to remove crude oils adhered to mineral particles. A molecular dynamics model was built to investigate the competitive adsorption process of various surfactants on diverse mineral surfaces, specifically in the presence of crude oil. The model was constructed using the Materials Studio software (The software version number is 20.1.0.2728) produced by Accelrys, Inc. (San Diego, CA, USA). The model is shown in Figure 13.

The model used crude oil as the dispersed phase due to its strong adsorption to rock minerals. The crude oil is adsorbed onto the mineral surface, while the surfactant solution serves as the exterior phase. The study focuses on the competitive adsorption of a solution containing crude oil and surfactant on various mineral surfaces. Given the intricate nature of the components in crude oil, the crude oil fraction was simplified by using hexane as the primary component. Subsequently, the surfactant molecules were dispersed in the oil phase fraction along with 500 molecules of hexane, 500 molecules of the aqueous phase, and 20 molecules of each of the various surfactants. The lower side was designated as a distinct mineral surface. Structural modeling of various minerals was obtained from the latest American Mineralogist Crystal Structure Database. The density distribution and MSD of crude oil molecules were used to describe the adsorption between various minerals and crude oil in the presence of surfactant molecules. The MSD can be expressed by Equation (4).

(4) $$MSD(\Delta t)={\left[\vec{\Delta r}(\Delta t)\right]}^{2}=\frac{1}{N}\sum_{i=1}^{N}{\left[{\vec{r}}_{i}(t+\Delta t)-{\vec{r}}_{i}(t)\right]}^{2}$$

where *N* is the number of particles, and ${\vec{r}}_{i}(t)$ is the coordinate of particle *i* at time *t*. *MSD* is to find the average square displacement at a specific time interval during the traversal time, Å^2^. The entire system undergoes an energy minimization process to create the initial model, which relies on the Lennard-Jones potential for intermolecular interactions, with a truncation radius of 12.5 Å. The Coulomb force is computed with the Particle–Particle–Particle–Mesh (PPPM) technique. Following the construction of the initial model, the shake method was employed to induce vibrations in the crude oil components, causing them to oscillate about their respective geometric positions. The entire system underwent kinetic simulation in the NVT ensemble, with a simulation duration of 1 nanosecond. The temperature of the system was controlled by a Nose—Hoover thermostat at 293.15 K with a time interval of 0.1 ps.

## 4. Conclusions

In summary, the process by which surfactant systems strip surface crude oils from minerals of varying grain sizes in sand-conglomerate has been elucidated. Surfactant systems can be effectively adsorbed onto the rock surface, changing the wettability of the rock surface to fluid in the formation, thereby increasing the hydrophilicity of the rock surface. This prompts the original adsorption of the crude oil onto the rock surface to gradually contract and aggregate, eventually stripping it off from the surface of the rock and facilitating transportation. Various minerals possess distinct electrical properties on their surfaces. When exposed to surfactants, rocks containing montmorillonite exhibit significant adsorption of surfactant, causing the desorption of crude oil and a decrease in the density of crude oil molecules on the rock surface. In conglomerate reservoirs, there exists a specific correlation between the mineral composition of the pore throat, where the crude oil is situated, and the surfactant system employed in the drive mechanism. If the surfactant has a higher affinity for the minerals, it increases the possibility of competitive adsorption and subsequent stripping of the crude oil.

## Data Availability

The data presented in this study are available upon request from the corresponding author.

## References

[1] Dong X., Liu H., Wang C., Lu C., Yan F. (2016). The polymer-enhanced foam injection process: An improved-oil-recovery technique for light oil reservoirs after polymer flooding. Energy Sources Part A.

[2] Yang E.L. (2011). The Application of Fuzzy Clustering Method in the Division of Reservoir Flow Unit. Adv. Mater. Res..

[3] Thakur G. (2019). Enhanced recovery technologies for unconventional oil reservoirs. J. Pet. Technol..

[4] Zhou Y., Wu S., Li Z., Zhu R., Xie S., Jing C., Lei L. (2018). Multifractal study of three-dimensional pore structure of sand-conglomerate reservoir based on CT images. Energy Fuels.

[5] Yu Z., Wang Z., Fan W., Wang J., Li Z. (2022). Evaluating the sedimentological and diagenetic impacts on terrestrial lacustrine fan delta sandy conglomerates reservoir quality: Insights from the Triassic Baikouquan Formation in the Mahu sag, Junggar Basin, Western China. Mar. Pet. Geol..

[6] Yin S., Chen Y., Wu X. (2018). Different pore structure modalities in sandy conglomerate reservoirs and their forming mechanisms. Arabian J. Geosci..

[7] Zhou Y., Wu S., Li Z., Zhu R., Xie S., Zhai X., Lei L. (2021). Investigation of microscopic pore structure and permeability prediction in sand-conglomerate reservoirs. J. Earth Sci..

[8] Gong Q., Liu Z., Zhu C., Wang B., Jin Y., Shi Z., Wu J. (2023). Heterogeneity of a Sandy Conglomerate Reservoir in Qie12 Block, Qaidam Basin, Northwest China and Its Influence on Remaining Oil Distribution. Energies.

[9] Ge X., Fan Y., Zhu X., Chen Y., Li R. (2015). Determination of nuclear magnetic resonance T_2_ cutoff value based on multifractal theory—An application in sandstone with complex pore structure. Geophysics.

[10] Ding L., Wu Q., Zhang L., Guérillot D. (2020). Application of Fractional Flow Theory for Analytical Modeling of Surfactant Flooding, Polymer Flooding, and Surfactant/Polymer Flooding for Chemical Enhanced Oil Recovery. Water.

[11] Mohsenatabar Firozjaii A., Derakhshan A., Shadizadeh S.R. (2018). An investigation into surfactant flooding and alkaline-surfactant-polymer flooding for enhancing oil recovery from carbonate reservoirs: Experimental study and simulation. Energy Sources Part A.

[12] Demirbas A., Alsulami H.E., Hassanein W.S. (2015). Utilization of surfactant flooding processes for enhanced oil recovery (EOR). Pet. Sci. Technol..

[13] Zhao Y., Sun Y., Liu S., Wang K., Jiang Y. (2017). Pore structure characterization of coal by NMR cryoporometry. Fuel.

[14] Lu J., Weerasooriya U.P., Pope G.A. (2014). Investigation of gravity-stable surfactant floods. Fuel.

[15] Bagrezaie M.A., Pourafshary P. (2015). Improvement of surfactant flooding performance by application of nanoparticles in sandstone reservoirs. J. Jpn. Pet. Inst..

[16] Jouenne S. (2020). Polymer flooding in high temperature, high salinity conditions: Selection of polymer type and polymer chemistry, thermal stability. J. Pet. Sci. Eng..

[17] Cheng Q., Cao G., Bai Y., Zhu Z., Zhang N., Li D. (2023). Probing the demulsification mechanism of emulsion with SPAN series based on the effect of solid phase particles. Molecules.

[18] Raffa P., Broekhuis A.A., Picchioni F. (2016). Polymeric surfactants for enhanced oil recovery: A review. J. Pet. Sci. Eng..

[19] Cao G., Du T., Bai Y., Yang T., Zuo J. (2021). Effects of surfactant molecular structure on the stability of water in oil emulsion. J. Pet. Sci. Eng..

[20] Afolabi F., Mahmood S.M., Yekeen N., Akbari S., Sharifigaliuk H. (2022). Polymeric surfactants for enhanced oil recovery: A review of recent progress. J. Pet. Sci. Eng..

[21] Cao G., Cheng Q., Liu Y., Bu R., Zhang N., Wang P. (2022). Influencing factors of surfactant stripping crude oil and spontaneous imbibition mechanism of surfactants in a tight reservoir. ACS Omega.

